# A Longitudinal Study of the Relation between Childhood Activities and Psychosocial Adjustment in Early Adolescence

**DOI:** 10.3390/ijerph18105299

**Published:** 2021-05-16

**Authors:** Rosa S. Wong, Keith T. S. Tung, Nirmala Rao, Frederick K. W. Ho, Ko Ling Chan, King-Wa Fu, Winnie W. Y. Tso, Fan Jiang, Jason C. S. Yam, David Coghill, Ian C. K. Wong, Patrick Ip

**Affiliations:** 1 Department of Paediatrics and Adolescent Medicine, The University of Hong Kong, Hong Kong, China; rosawg@connect.hku.hk (R.S.W.); keith-tung@connect.hku.hk (K.T.S.T.); wytso@hku.hk (W.W.Y.T.); 2 Centre for Safe Medication Practice and Research, Department of Pharmacology and Pharmacy, The University of Hong Kong, Hong Kong, China; wongick@hku.hk; 3 Faculty of Education, The University of Hong Kong, Hong Kong, China; nrao@hku.hk; 4 Institute of Health and Wellbeing, University of Glasgow, 1 Lilybank Gardens, Glasgow G12 8RZ, UK; Frederick.Ho@glasgow.ac.uk; 5 Department of Applied Social Sciences, The Hong Kong Polytechnic University, Hong Kong, China; koling.chan@polyu.edu.hk; 6 Journalism and Media Studies Centre, The University of Hong Kong, Hong Kong, China; kwfu@hku.hk; 7 Department of Developmental and Behavioral Pediatrics, Shanghai Children’s Medical Center, Shanghai Jiao Tong University School of Medicine, Shanghai 200240, China; riversailer@hotmail.com; 8 Ministry of Education-Shanghai Key Laboratory of Children’s Environmental Health, Shanghai 200092, China; 9 Department of Ophthalmology and Visual Sciences, The Chinese University of Hong Kong, Hong Kong, China; yamcheuksing@cuhk.edu.hk; 10 Department of Paediatrics and Psychiatry, Faculty of Medicine, Dentistry and Health Sciences, University of Melbourne, Melbourne, VIC 3010, Australia; David.Coghill@rch.org.au; 11 Murdoch Children’s Research Institute, Melbourne, VIC 3052, Australia; 12 Research Department of Practice and Policy, UCL School of Pharmacy, London WC1E 6BT, UK

**Keywords:** psychosocial development, adolescence, early-life activities, screen time, cohort study

## Abstract

Background: Although an increasing body of research shows that excessive screen time could impair brain development, whereas non-screen recreational activities can promote the development of adaptive emotion regulation and social skills, there is a lack of comparative research on this topic. Hence, this study examined whether and to what extent the frequency of early-life activities predicted later externalizing and internalizing problems. Methods: In 2012/13, we recruited Kindergarten 3 (K3) students from randomly selected kindergartens in two districts of Hong Kong and collected parent-report data on children’s screen activities and parent–child activities. In 2018/19, we re-surveyed the parents of 323 students (aged 11 to 13 years) with question items regarding their children’s externalizing and internalizing symptoms in early adolescence. Linear regression analyses were conducted to examine the associations between childhood activities and psychosocial problems in early adolescence. Results: Early-life parent–child activities (β = −0.14, *p* = 0.012) and child-alone screen use duration (β = 0.15, *p* = 0.007) independently predicted externalizing problems in early adolescence. Their associations with video game exposure (β = 0.19, *p* = 0.004) and non-screen recreational parent–child activities (β = −0.14, *p* = 0.004) were particularly strong. Conclusions: Parent–child play time is important for healthy psychosocial development. More efforts should be directed to urge parents and caregivers to replace child-alone screen time with parent–child play time.

## 1. Introduction

Behavioral problems can be understood as internalizing and externalizing problems. Internalizing problems generally refer to emotional symptoms such as anxiety and depression, whereas externalizing problems are observable rule-breaking behavior such as delinquency and aggression. These problems often persist into adulthood and increase one’s susceptibility to mental disorders [1]. For example, childhood behavioral problems have been found to be associated with high-risk behaviors (e.g., substance use) during adolescence [2,3]. Although numerous studies have documented the impact of early home environment on children’s academic and language skills [4,5,6,7], only a handful of studies have examined the long-term consequences of exposure to different types of home environment risks such as limited learning materials or low parental responsiveness and involvement [8,9]. Evidence about the relationship between early life exposures at home and later behavioral outcomes in adolescence is even more scant. Notably, screen use and parent–child interactions are increasingly viewed as important factors that may influence the developmental trajectories of externalizing and internalizing symptoms. However, existing studies mainly use cross-sectional design and focus on the association of a single type of activity (e.g., screen time or parent–child interactions) to a later outcome. There has been a dearth of research on the independent and combined predictive effects of these activities on long-term health and behaviors. 

In recent years, with the increasing use of screen media within families and the rapid growth and development of digital learning and entertainment, there have been concerns about the short- and long-term consequences of early screen exposure [10]. Emerging evidence from cross-sectional studies shows that excessive screen time such as watching TV or playing videogames is associated with a wide range of problems across multiple domains including mood disorders [11], engagement in multiple risk behaviors [12], sleeping difficulties [13,14], and increased sedentary time [15]. There is also longitudinal evidence, albeit limited, indicating the association between changes in recreational screen-time and changes in mental health outcomes [16], whereas another longitudinal study reported no association between screen time at early age and attention deficit hyperactivity disorder (ADHD) later in life [17]. However, Carson, Pickett, and Janssen (2011) found that compared to television and video game use, high computer use showed stronger association with multiple risk behaviors in both boys and girls [12]. In a longitudinal study of Australian children, total screen time was found to be associated with poor temperament and health problems, although the pattern of associations varied across types of screen use [18]. 

From another perspective, research findings have consistently demonstrated the benefits of sufficient, high-quality parent–child interactions in early childhood for the development of adaptive social, emotional, and cognitive skills later in life. It has been posited that any effects due to parent–child relationship can be understood through the quality of parenting and parent–child interactions [19]. An earlier study reported that rigidity in parent–child interactions, such as not participating in play activities with children, was associated with increased levels of internalizing and externalizing symptoms in childhood [20]. Recent studies on technoference (the interruptions in interpersonal communication caused by attention paid to personal technological devices) also provide evidence that technologies-mediated interruptions to parent–child interactions can negatively affect children’s perceptions of parental warmth and their psychosocial wellbeing [21,22]. Furthermore, different types of parent–child activities such as learning or recreational activities might have different implications for social-emotional functioning [23]. This line of evidence suggests that examining the influences of overall and specific types of parent–child activities, whether they are learning- or recreational-based, could provide insights into children’s developmental outcomes. 

Although findings concerning the behavioral consequences of excessive screen use and insufficient parent–child interactions are available, research examining the relative importance of early-life screen use and non-screen parent–child activities such as reading and storytelling for long-term development is lacking. Hence, the purpose of this study was to explore whether and to what extent the frequency of early-life activities (non-screen parent–child activities and screen activities) predicted later externalizing and internalizing problems. More specifically, we addressed the following questions: (a) Do the amount of screen use by children and the frequency of non-screen parent–child activities independently predict behavioral outcomes longitudinally, after controlling for confounding variables reported in previous literature (e.g., child characteristics and family socioeconomic status (SES)) [24]? (b) Do associations between screen use and behavioral problems vary as a function of the type of screen use (e.g., video game, television, and computer use)? Finally, (c) do associations between non-screen parent–child activities and behavioral problems vary as a function of the type of activity (e.g., recreational and learning activities)? Based on extant research [10,14,22,24,25], we hypothesized that higher frequency of non-screen parent–child activities, particularly for recreational activities, would predict fewer behavioral problems; in contrast, screen time, particularly for time spent on video games, would predict more behavioral problems. 

## 2. Method

### 2.1. Study Design

Analyses were performed using the data collected in the prospective HealthyKids cohort study. The HealthyKids cohort study was originally designed to examine the association between family socioeconomic position in early life and health conditions at older ages [26]. Study participants were recruited from 20 randomly selected kindergartens in an affluent district (Hong Kong Island) and a less wealthy district (Yuen Long) of Hong Kong. Parents provided written informed consent for themselves and on behalf of their children. Data were collected through direct assessment and questionnaires. In the present study, data from baseline assessment (2011–2012) and 7-year follow-up (2018–2019) were used. Standardized and validated measures were used to assess demographic characteristics, child-alone screen activities, parent–child activities, and behavioral problems across time points. The study protocol was approved by the Institutional Review Board of The University of Hong Kong/Hospital Authority Hong Kong West Cluster (UW 18-057).

### 2.2. Participants

During the baseline assessment, 502 parents of final-year preschoolers in 20 kindergartens randomly selected from two districts of Hong Kong provided data on family demographics, time spent by children on electronic devices, and frequency of parent–child activities. Their children’s socioemotional skills as indicators of early-life internalizing and externalizing problems were also assessed by teachers using the Chinese version of the Early Development Instrument [26,27]. For the present study, longitudinal data collected over a period of seven years (at baseline (time 1) and 7-year follow-up (time 2)) were used. We invited families who provided data at time 1 to participate in the follow-up study at time 2 by phone. Parents who expressed interest to join this study were then sent with a set of questionnaires concerning the variables of interest in this study. They were welcomed to call or text the research team if they encountered difficulties in completing the questionnaire items. The parents were also instructed to mail their completed questionnaire to the research team office. Consequently, this study analyzed data from 323 adolescents after excluding those with missing data on the externalizing and internalizing problems at the 7-year follow-up (*n* = 179 including 71 with no valid contact, 22 with no data on the behavioral outcomes of interest, and 86 refusing to participate in this follow-up survey). Table 1 displays sociodemographic characteristics of the study sample at time 1 (mean age: 4.43 years). Their average monthly household income was HKD 44,600 (USD 5718). Over half of the fathers and mothers had completed at least upper secondary education. Compared to those not in our analyses due to missing data, the analytic sample included those with higher parental baseline socioeconomic position (SES: 0.11 vs. −0.11; *t* (490) = 2.33, *p* = 0.022); the samples were otherwise similar in terms of children’s age, gender, and socioemotional competence at time 1. 

## 3. Measures

### 3.1. Parent-Child Activities

Parent–child activities were assessed by the Chinese Parent–Child Interaction Scale (CPCIS, [28]) at time 1. The CPCIS was completed by the parent at time 1 to assess the weekly frequency of parent–child activities (arithmetic/mathematics, English alphabet, Chinese characters, reading, drawing, singing, storytelling, and discussing news and current affairs). These activities can be subdivided into learning activities and recreational activities subscales [26]. Items were rated on a 4-point scale ranging from 0 = none to 3 = 4 times or above per week and averaged to generate an overall (M = 1.93, SD = 0.53) and domain-specific mean score, with higher scores indicating more frequent parent–child activities. The CPCIS has been validated using the data collected in the kindergarten phase of the HealthyKids cohort study, and demonstrated adequate construct validity and good internal consistency (α = 0.71 for recreational activities and 0.78 for learning activities) [28].

### 3.2. Screen Time

Children’s screen time was reported by the parent at time 1 by completing a screen time questionnaire designed to assess time spent watching television, video gaming on handheld and/or other types of game consoles and using computers, tablet computers and smartphones for studying, gaming and/or web browsing. The average amount of daily time spent on overall (M = 2.16 h, SD = 1.67) and specific screen time types (watching television, computer use for non-video game activities, and video gaming) was calculated by averaging the amount of time spent on that particular screen activity for both weekends and weekdays using the weighted average formula ([2 × weekend + 5 × weekday] ÷ 7) and reported in hours.

### 3.3. Externalizing and Internalizing Problems

Parents also completed the Strength and Difficulties Questionnaire (SDQ) [29] at time 2. It has 25 items assessing conduct problems, hyperactivity, emotional problems, peer problems and prosocial behavior. Its Chinese version has been widely used in preschooler research in Hong Kong [30]. The conduct problems and hyperactivity scales were summed to derive an externalizing score, whereas the emotional and peer problems scales were summed to generate an internalizing score. The internalizing and externalizing behavior subscales all showed good internal reliability coefficients (α = 0.70 and 0.77, respectively) for this sample. Furthermore, their convergent validity was supported by the significant correlation between the internalizing behavior subscale and the adolescent self-report version of the Depression Anxiety Stress Scale—21 (DASS-21) scores (absolute values of r from 0.22–0.32, *p* < 0.001), whereas the externalizing behavior subscale score was significantly higher in adolescents with a diagnosis of ADHD recorded in the hospital system (7.80 vs. 4.80, *t* (313) = 3.72, *p* < 0.001). 

### 3.4. Covariates

In addition, the following relevant covariates (identified based on literature) had available data in the HealthyKids cohort study and would be included in subsequent analyses: demographics (age, gender, and a family socioeconomic status index incorporating maternal and paternal educational attainment, maternal and paternal occupation, family monthly income adjusted for household size, and family asset score [26]) and time 1 socio-emotional readiness (using the Chinese version of Early Development Instrument) [27].

### 3.5. Data Analysis

Descriptive statistics including frequencies and percentages or means and standard deviations were calculated to characterize the cohort. All continuous variables were confirmed with normal distribution by normality measures (skewness and kurtosis) and visual inspection of plots. Multiple linear regression models tested each of the hypotheses concerning externalizing and internalizing problems using a hierarchical approach. To explore the overall activity effect, the first model included an overall activity category variable (parent–child activities or screen time) as a predictor. The second model examined its independent effect by controlling for the effect of the other overall activity category. The third model built upon the second model with the addition of age, gender, family SES and socioemotional readiness assessed at time 1 as covariates. To examine the effect of each activity type, the first model included the activity of interest as a predictor. The second model included the activity variable and other activity types to test their independent effects. In the third model, apart from the activity variables, the covariates (age, gender, family SES and socioemotional readiness assessed at time 1) were added. Lastly, a fourth model was calculated by adjusting for the other overall activity category in the third model. All regression coefficients (β) were standardized with β = 0.1 as small, β = 0.3 as medium and β = 0.5 as large according to the guidelines by Cohen [31]. Prior to model calculations, we replaced missing data of predictors and control variables using the multiple imputation by chained equations (MICE) R package [32] to include all cases (*n* = 323). Missing values were below 5% for all predictors. In addition, a sensitivity analysis was computed to confirm whether results with and without imputation were the same. All data were analyzed using R statistical software (version 3.5.1, 2 July 2018, R Foundation for Statistical Computing, Vienna, Austria) with *p* < 0.05 indicating statistical significance for all analyses. 

## 4. Results

Table 2 presents the longitudinal associations of parent–child activities and children’s screen time at time 1 (as unique predictors) with externalizing and internalizing problems in early adolescence. More externalizing problems at time 2 were significantly associated with fewer parent–child activities (β = −0.14, *p* = 0.012) and more time spent by children on screen activities (β = 0.15, *p* = 0.007) at time 1. However, after further controlling for children’s gender, age, and time 1 socioemotional competence and family SES, the association between parent–child activities and later externalizing problems became marginally significant, whereas the association between screen time and later externalizing problems remained significant (β = 0.12, *p* = 0.038). Neither parent–child activities nor screen time was associated with internalizing problems in the early adolescent sample.

To further illustrate the relationship between each activity type and later behavioral problems, we tested regression models with each screen or parent–child activity as a separate, unique predictor of subsequent internalizing and externalizing problems. As shown in Table 3, none of the different types of screen exposures were predictive of time 2 internalizing problems, although the predictive power of video game exposure (β ranging from 0.11 to 0.15) was larger than the other two exposures (television: β ranging from −0.01 to 0.06; computer: β ranging from 0.03 to 0.05). On the other hand, even after controlling for other types of screen exposure and parent–child activities at time 1 and sociodemographic characteristics, video game exposure was significantly associated with more externalizing problems (β = 0.19, *p* = 0.004) at time 2. This association was not observed when the predictor was television or computer use, suggesting that compared to television and computer use, time spent on video gaming was a more robust predictor of externalizing problems in this sample. 

In terms of parent–child activities (Table 4), neither learning (β ranging from −0.02 to −0.001) nor recreational activities (β ranging from −0.08 to 0.002) at time 1 were predictive of time 2 internalizing problems and their power were small. When comparing the predictive power of learning and recreational activities, recreational activities were a stronger predictor of time 2 externalizing problems (learning: β = 0.04, *p* = 0.508 vs. recreational: β = −0.14, *p* = 0.004). This suggests that compared to learning activities, recreational interactions between parents and children in early years had a stronger association with later externalizing problems. 

## 5. Discussion

This study examined the extent to which the duration of children’s exposure to screen and the frequency of non-screen parent–child activities longitudinally predicted externalizing and internalizing problems in early adolescence, controlling for child and family characteristics. Consistent with our hypothesis, we found that spending more time on non-screen parent–child activities and less time on child-alone video game activities predicted fewer externalizing problems in early adolescence. Prior reports have shown that children who spent more time on electronic devices were found to have more externalizing problems at an early age [25], and our findings add to the literature by demonstrating the long-term behavioral consequences of early screen time use. Interestingly, the effect of early parent–child activities on externalizing problems was attenuated after family SES and children’s level of social–emotional school readiness were held constant. This finding suggests that the effect and nature of parent–child activities may vary considerably across socioeconomic strata. Children’s characteristics, such as temperament and personal interests, may also be factors to consider when planning activities for them. Although the associations with internalizing problems are generally weaker, the results point to the same direction that internalizing problems could be mitigated with increased parent–child activities and exacerbated with increased screen activities. 

Previous research reporting no association between screen time and externalizing behavior mainly focused on overall screen use duration, but did not differentiate the effect of different types of screen activities [17]. Results from this study provide new insights suggesting that different types of screen activities can have different effects on children’s behavior. The echoes the notion reported in previous studies [12,18]. Specifically, by examining the relative effect of video game, television, and computer use in parallel, the present study found that video game use in childhood was most strongly associated with externalizing problems in early adolescence. This could be partly explained by children imitating the on-screen violent behavior that is contained in many video games [33]. The other possible reason could be that the overstimulating and arousing game content may delay bedtime and desensitize children to other enriching non-screen activities such as reading and outdoor-play [10]. Our findings suggest that it is important for parents to control children’s screen use behavior and limit developmentally inappropriate screen content, particularly in early childhood when the brain is most susceptible to environmental exposures [34]. However, the evidence as to which type of screen use behavior is the most problematic for health and development has been mixed. For instance, a 12-month prospective study examining television, computer, and video game use found that high computer use was the strongest predictor of multiple risk behaviors in grades 9–10 youth [12], whereas a large-scale cross-sectional study comparing the health effects of television, computer, and combined media use found larger effects from television viewing [35]. These inconsistent findings could be due to methodological differences such as variable target outcomes and measures across studies. Nonetheless, overall evidence supports the notion that excessive screen time could have adverse effects on children and adolescents. 

In the comparison of different types of parent–child activities, early recreational activities, not learning activities, were found to protect against externalizing problems even after controlling for the effects of electronic device use. These differences could be partly attributed to the parent–child interaction behavior that occurs during the activity process. As highlighted in the literature, the unstructured style of recreational activities provides plenty of free-play time for open-ended and imaginative exploration. During these activities, parents can teach children how to regulate their emotions and behavior, which will likely enhance their adjustment to different situations and social interactions later in life [24]. As such, it is possible that children exposed to recreational activities would have greater behavioral control and exhibit fewer externalizing problems. On the other hand, learning activities are mostly structured and follow a pre-designed syllabus based on school curriculum needs and requirements. Compared to recreational activities, these structured academic activities might give children fewer opportunities to explore feelings in self and others, and thus have smaller effects on subsequent behavioral outcomes. Consistent with previous research [36,37], this study also demonstrates a need to develop comprehensive parenting programs aiming to train parents on multiple aspects of parenting such as guiding them to provide sensitive and responsive care as well as assisting them to establish positive interactions with their children through different activities. 

This 7-year longitudinal study allowed for the examination of various types of early-life activities and their role in the development of internalizing and externalizing behavioral problems over a long period of time. However, there are several limitations that should be addressed in future research. First, all the measures in this study were completed by parents, which may introduce a certain degree of reporting bias. Although the parent-reported SDQ scores showed good convergent validity with the adolescents self-reported DASS-21 scores and ADHD diagnosis in this study, future research should collect and combine ratings from multiple informants such as teachers or both parents to enhance the reliability of the results. Second, we measured only the frequency of activities, but did not examine the quality of parent–child interaction. In addition, the lack of relevant data made us unable to confirm whether parents and children playing electronic devices together would show differential effects and which type of video games the children played at an early age. Future research would benefit from more comprehensive measures to assess the types and frequency of these activities and the gestures involved during the event. Third, these findings may not be generalizable to all families because of the potential risk of attrition bias. Similar to the limitations of other longitudinal studies [38], our analytic sample also tended to be relatively higher in SES. Future research should explore strategies to engage participants with lower SES in follow-up study phases. 

## 6. Conclusions

The present study offers novel insights about the predictive power of different types of early childhood activities (screen versus non-screen) for the development of behavioral problems in early adolescence. In particular, early screen exposure appears to have a strong and negative effect on future behavioral outcomes. It should be noted that the behavioral consequences of early screen use are difficult to undo and cannot be remedied by regular parent-child interactions and engagement in play-based activities together. Our findings suggest a need for broader parenting initiatives with training on adaptive parenting skills and activity planning strategies as targets for intervention. Teachers and health professionals working with children and families should also educate parents about the harmful effects of early screen use and offer families suggestions and resources on alternative activities that would favor a child’s mental, social, and emotional development to a greater extent. 

## Figures and Tables

**Table 1:** Subject characteristics.

	Overall (*N* = 323)
	Mean (SD)/*n* (%)
Age	4.43 (2.08)
Family socioeconomic status index (Time 1)	0.11 (0.99)
Time 1 monthly household income (HKD ′000)	44.60 (29.57)
Education attainment of father	
Junior secondary school and below	80 (24.8)
Senior secondary school to associate degree	112 (34.7)
Bachelor’s degree or above	124 (38.4)
Missing	7(2.2)
Education attainment of mother	
Junior secondary school and below	82 (25.4)
Senior secondary school to associate degree	123 (38.1)
Bachelor’s degree or above	118 (36.5)
CPCIS parent–child activities (Time 1)	1.93 (0.53)
CPCIS learning activities	2.03 (0.64)
CPCIS recreational activities	1.86 (0.60)
Total screen time (hour/day) (Time 1)	2.16 (1.67)
Time spent on TV (hour/day)	1.20 (1.02)
Time spent on game (hour/day)	0.70 (0.89)
Time spent on PC (hour/day)	0.27 (0.43)
CEDI socioemotional readiness (Time 1)	8.33 (1.31)
SDQ (Time 2)	
Internalizing problem score	4.18 (3.10)
Externalizing problem score	4.94 (3.11)

CPCIS: Chinese Parent–Child Interaction Scale; CEDI: Chinese Early Development. Index; SDQ: Strength and Difficulties Questionnaire.

**Table 2:** Longitudinal effect of parent–child and child screen activities on later externalizing and internalizing problems.

	Externalizing Problems		Internalizing Problems	
	Parent-Child Activities	Time Spent on Electronic Devices	Parent-Child Activities	Time Spent on Electronic Devices
	β (95% CI, *p*-value)	β (95% CI, *p*-value)	β (95% CI, *p*-value)	β (95% CI, *p*-value)
Univariable	−0.18 (−0.29 to −0.07, *p* = 0.001)	0.19 (0.08 to 0.30, *p* < 0.001)	−0.07 (−0.18 to 0.04, *p* = 0.225)	0.14 (0.00 to 0.27, *p* = 0.050)
Bivariable ^a^	−0.14 (−0.26 to −0.03, *p* = 0.012)	0.15 (0.04 to 0.26, *p* = 0.007)	−0.04 (−0.15 to 0.08, *p* = 0.504)	0.13 (−0.01 to 0.27, *p* = 0.074)
Adjusted for confounders ^b^	−0.11 (−0.23 to 0.00, *p* = 0.051)	0.12 (0.01 to 0.23, *p* = 0.038)	0.003 (−0.11 to 0.12, *p* = 0.957)	0.09 (−0.04 to 0.22, *p* = 0.169)

^a^ In the bivariable model, we adjusted for time 1 CPCIS parent–child activities and child time spent on electronic devices to test the independent associations. ^b^ Bivariable model (a) adjusted for age and gender, family socioeconomic status, and CEDI socio-emotional readiness assessed at time 1.

**Table 3:** Effect of specific screen activity type on later externalizing and internalizing problems.

	Externalizing Problems			Internalizing Problems		
	Game	TV	PC	Game	TV	PC
	β (95% CI, *p*-value)	β (95% CI, *p*-value)	β (95% CI, *p*-value)	β (95% CI, *p*-value)	β (95% CI, *p*-value)	β (95% CI, *p*-value)
Univariable	0.25 (0.14 to 0.36, *p* < 0.001)	0.10 (−0.01 to 0.21, *p* = 0.068)	−0.08 (−0.20 to 0.03, *p* = 0.151)	0.15 (−0.03 to 0.34, *p* = 0.086)	0.06 (−0.06 to 0.18, *p* = 0.328)	0.03 (−0.08 to 0.15, *p* = 0.550)
Multivariable ^a^	0.25 (0.14 to 0.37, *p* < 0.001)	0.01 (−0.10 to 0.13, *p* = 0.852)	−0.10 (−0.21 to 0.02, *p* = 0.091)	0.15 (−0.06 to 0.36, *p* = 0.143)	0.006 (−0.14 to 0.15, *p* = 0.935)	0.03 (−0.08 to 0.14, *p* = 0.630)
Adjusted for confounders ^b^	0.22 (0.09 to 0.34, *p* < 0.001)	−0.006 (−0.12 to 0.11, *p* = 0.917)	−0.08 (−0.19 to 0.03, *p* = 0.162)	0.10 (−0.08 to 0.28, *p* = 0.234)	−0.01 (−0.14 to 0.12, *p* = 0.857)	0.05 (−0.06 to 0.16, *p* = 0.365)
Adjusted for parent–child activities ^c^	0.19 (0.06 to 0.32, *p* = 0.004)	−0.02 (−0.14 to 0.10, *p* = 0.739)	−0.07 (−0.18 to 0.04, *p* = 0.235)	0.11 (−0.08 to 0.29, *p* = 0.233)	−0.01 (−0.14 to 0.12, *p* = 0.859)	0.05 (−0.06 to 0.16, *p* = 0.360)

^a^ In the bivariable model, we adjusted for time 1 child time spent on game, TV and PC to test independent associations. ^b^ Bivariable model (a) adjusted for age and gender, family socioeconomic status, and CEDI socio-emotional readiness assessed at time 1. ^c^ Adjusted bivariable model (b) further adjusted for parent–child learning and recreational activities at time 1.

**Table 4:** Effect of specific parent–child activity type on later externalizing and internalizing problems.

	Externalizing Problems		Internalizing Problems	
	Learning Activities	Recreational Activities	Learning Activities	Recreational Activities
	β (95% CI, *p*-value)	β (95% CI, *p*-value)	β (95% CI, *p*-value)	β (95% CI, *p*-value)
Univariable	−0.05 (−0.16 to 0.06, *p* = 0.368)	−0.22 (−0.33 to −0.11, *p* < 0.001)	−0.03 (−0.14 to 0.08, *p* = 0.564)	−0.08 (−0.19 to 0.04, *p* = 0.186)
Multivariable ^a^	0.05(−0.07 to 0.17, *p* = 0.427)	−0.24 (−0.36 to −0.12, *p* < 0.001)	−0.001 (−0.12 to 0.12, *p* = 0.984)	−0.07 (−0.20 to 0.05, *p* = 0.235)
Adjusted for confounders ^b^	0.03 (−0.09 to 0.16, *p* = 0.610)	−0.19 (−0.32 to −0.05, *p* = 0.006)	−0.02 (−0.14 to 0.10, *p* = 0.701)	0.002 (−0.13 to 0.13, *p* = 0.969)
Adjusted for electronic device use ^c^	0.04 (−0.09 to 0.16, *p* = 0.508)	−0.14 (−0.28 to −0.004, *p* = 0.044)	−0.03 (−0.15 to 0.10, *p* = 0.655)	0.02 (−0.11 to 0.15, *p* = 0.746)

^a^ In the bivariable model, we adjusted for time 1 parent–child learning and recreational activities to test independent associations. ^b^ Bivariable model (a) adjusted for age and gender, family socioeconomic status, and CEDI socio-emotional readiness assessed at time 1. ^c^ Adjusted bivariable model (b) further adjusted for electronic device use at time 1.

## Data Availability

The data that support the findings of this study are available on request from the corresponding author.
